# Improving medical student recruitment into neurosurgery through teaching reform

**DOI:** 10.1186/s12909-022-03722-y

**Published:** 2022-09-02

**Authors:** Jun Shen, Lili Yuan, Ruixiang Ge, Xuefei Shao, Xiaochun Jiang

**Affiliations:** 1 Department of Neurosurgery, The First Affiliated Hospital of Wannan Medical College (YiJiShan Hospital of Wannan Medical College), Anhui Province 241001 Wuhu City, People’s Republic of China; 2 Department of Neurology, The First Affiliated Hospital of Wannan Medical College (YiJiShan Hospital of Wannan Medical College), Wuhu City, Anhui Province People’s Republic of China

**Keywords:** Case-based learning, Neurosurgery, Problem-based learning, Traditional teaching method

## Abstract

**Objective:**

This study aimed to determine whether a combination of case-based learning (CBL) and problem-based learning (PBL) methods in teaching can improve the academic performance and recruitment of medical students for neurosurgery.

**Methods:**

Four classes of fourth-year medical students were randomly divided into two groups. The traditional model group received the traditional teaching method, and the CBL-PBL group received the combined teaching methods of CBL and PBL. After the courses, the differences between the two groups in self-perceived competence, satisfaction with the course, post-class test scores, and clinical practice abilities were compared, and the proportions of neurosurgery major selection in pre- and post-curriculum between the two groups were also analyzed.

**Results:**

Self-perceived competence, post-class test scores, and clinical practice abilities in the CBL-PBL group were better than those in the traditional model group. The students in the CBL-PBL group showed a higher degree of satisfaction with the course than those in the traditional model group (χ2 = 12.03, *P* = 0.007). At the end of the semester, the proportion of students who chose neurosurgery majors in the CBL-PBL group was 13.3%, more than the 3.4% in the traditional model group (χ2 = 3.93, *P* = 0.048).

**Conclusion:**

Compared with the traditional teaching method, the CBL and PBL integrated method is more effective for improving the performance of medical students and enhancing their clinical capabilities in neurosurgery teaching. The CBL-PBL method effectively improved students’ interests in neurosurgery, potentially contributing to increasing medical student recruitment into neurosurgery.

**Supplementary Information:**

The online version contains supplementary material available at 10.1186/s12909-022-03722-y.

## Background

Neurosurgery must continue to attract the best and brightest talent to promote the continuous development and survivability of our profession [1]. The shortage of neurosurgeons in many countries has caused great social concern, which may lead to the lack of timely treatment and care of neurosurgical diseases, thus causing severe symptoms, disability, poor life quality of patients, and increasing the burden on families and society [2–4]. For such patients, families and healthcare center will pay substantial medical bills for maintenance treatment, nursing care, life support and rehabilitation. This further exacerbates the need for neurosurgeons, creating a vicious circle. Thus, the neurosurgeon shortage also will have financial effects on healthcare systems, promoting student recruitment into neurosurgery through education not only benefits for patients’ care, but also helps alleviate the financial burden on families and countries.

However, few medical students choose neurosurgery as a career in China. In postgraduate exams, in particular, neurosurgery is not usually the first choice of specialty, and some students were transferred to neurosurgery only when they were not accepted by other majors. There are several reasons for this phenomenon. First, neurosurgery requires comprehensive knowledge in the fields of neurology, anatomy, trauma care, critical care, and radiology [5], which is more difficult than other majors from the feedback of previous teaching effects. Moreover, only eight class hours are scheduled in neurosurgery teaching according to the arrangements of the Ministry of Education, and it is impossible for students to master the complicated knowledge of neurosurgery in such a short time. Second, the income of neurosurgeons is lower than that of other majors in China. The imbalance between income and risk hardly arouses students’ interests in the profession. Last, neurosurgery started late in China. Many county-level hospitals do not have neurosurgery departments, which makes it difficult for neurosurgery students to find jobs after graduation.

The traditional teaching method is regarded as a teacher-centered educational approach, emphasizing the delivery of the syllabus and knowledge points. The teacher explains the theoretical knowledge while the students receive the knowledge passively. This method has been widely used in medical and clinical teaching in the past few decades [6, 7]. However, the knowledge system of medical courses is complex and multifaceted [8, 9], which leads to students losing the motivation and the ability to self-learn. The combination of curriculum and clinical work is insufficient to develop students’ clinical cognition. Therefore, traditional teaching methods have become increasingly unsuitable for modern medical education and have been gradually replaced by other teaching methods [6, 10].

Case-based learning (CBL) is a patient-oriented and student-centered group discussion-style teaching approach, and problem-based learning (PBL) is an approach focused on the patients’ problem, engaging students in learning problem-solving skills. Both CBL and PBL approaches integrate theory into practice, cultivate students in self-guided learning, collaborate with fellows and scientific inquiry, and develop critical thinking, decision-making and clinical problem-solving abilities [11–16]. The CBL and PBL integrated method is more effective than traditional teaching methods in the teaching of various clinical specialties [17–19].

However, CBL and PBL integrated methods are rarely applied in neurosurgery teaching, even in neurosurgery training. In this study, we use the CBL and PBL integrated method in neurosurgery teaching to investigate whether students can benefit from teaching reforms that would change their perception of neurosurgery and increase their interest in neurosurgery.

## Students and methods

### Students

This was a prospectively randomized controlled study. We enrolled four classes of fourth-year medical students at the Wannan Medical College (30 students per class). The four classes were randomly divided into two groups. The traditional model group received the traditional teaching method, and the CBL-PBL group received the combined teaching method of CBL-and PBL. All students were anonymous and blinded to this study, which was represented by numbers.

### Study design

**Fig. 1:**
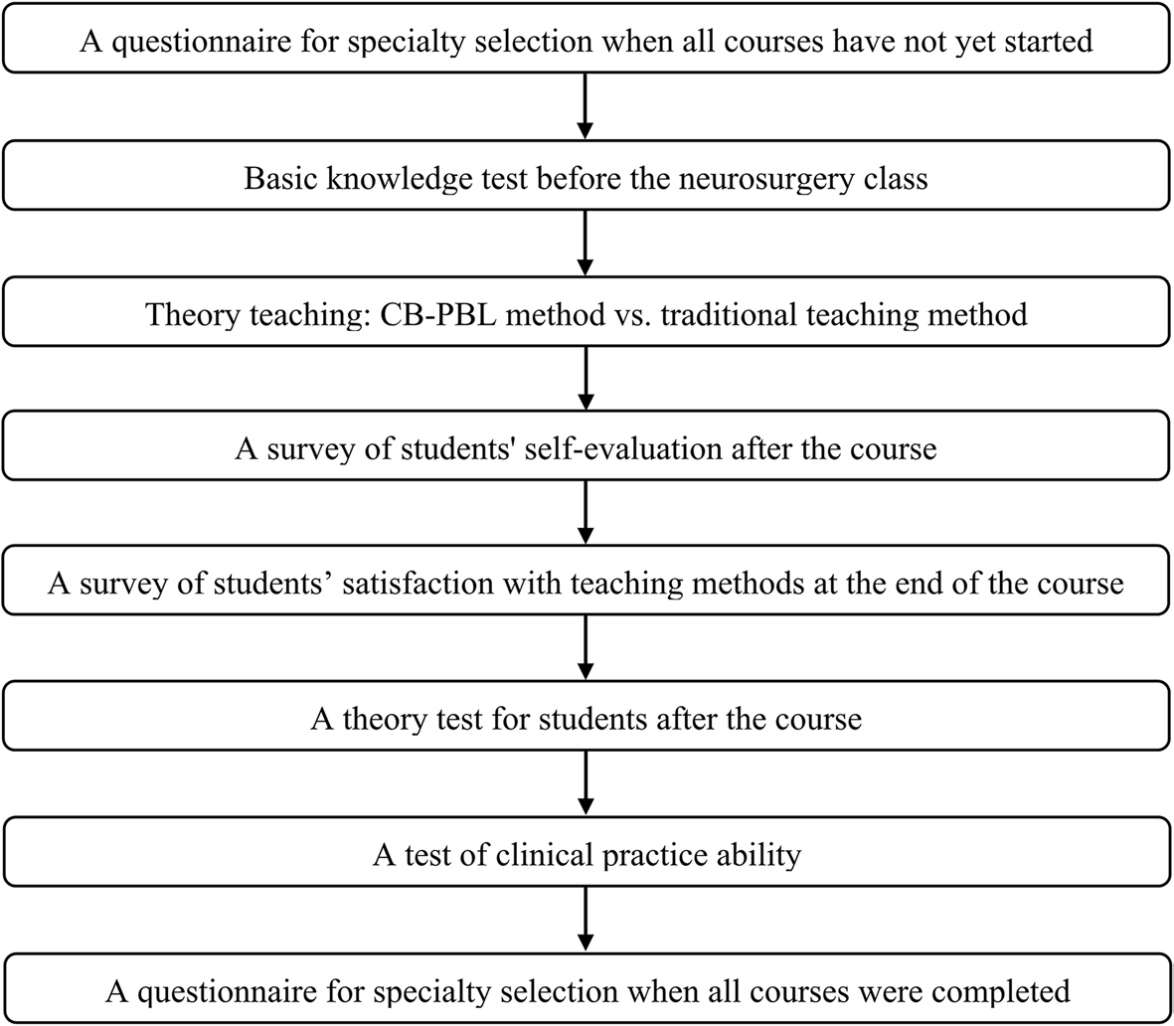
The experimental flow chart of this study

The experimental flow chart of this study is shown in Fig. 1. Before fourth-year students started the curriculum (internal medicine, surgery, obstetrics and gynecology, pediatrics, etc., were completed in the fourth year), we distributed a questionnaire of specialty selection to all students to understand the students’ interest in neurosurgery and arranged a basic knowledge test for them before the neurosurgery class.

The CBL-PBL teaching plan was implemented as follows. Before class, the teacher prepared a typical case and compiled the knowledge points into questions and then distributed the case and questions to the WeChat group class. The students were divided into 5 groups, and every group independently consulted textbooks, literature, and websites to determine the answers to the questions. In class, students formed a group discussion and reported the answers under the guidance of the teacher. After the student presentation, the teacher corrected them appropriately and summarized the knowledge points.

**Fig. 2:**
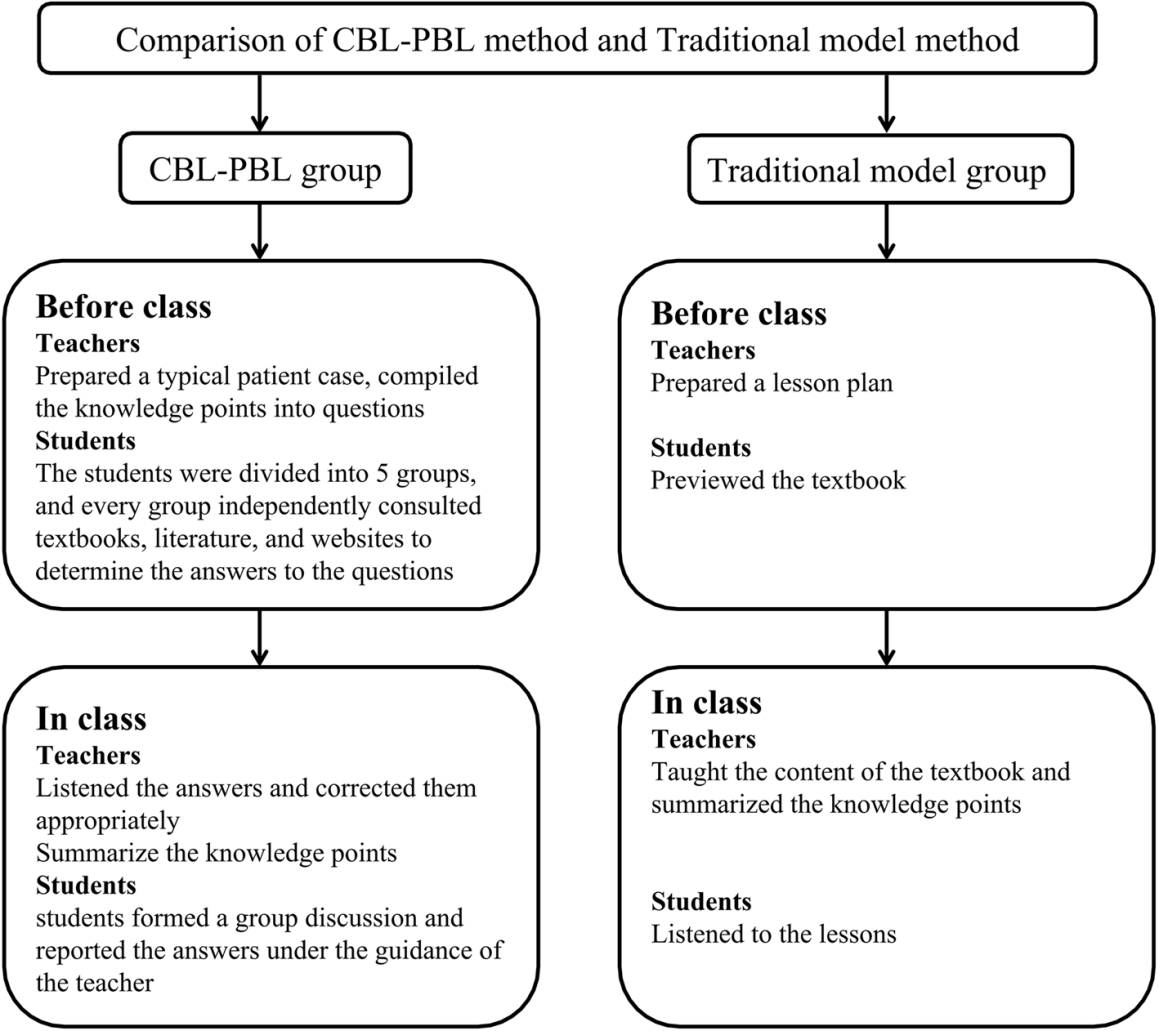
Comparison of CBL-PBL teaching and traditional teaching

The traditional teaching plan was implemented as follows. The teacher prepared a lesson plan and distributed the lesson plan to the WeChat group class. Students previewed the textbook according to the lesson plan. In class, the teacher explained the content of the textbook and summarized the knowledge points. Students listened to the teachers’ interpretation (Fig. 2).

After the course, we conducted a survey of students’ self-evaluation and their satisfaction with teaching methods and then arranged tests of theory and clinical practice abilities for the students. The clinical abilities test was completed in the neurosurgery ward. The teacher selected a typical case, and the students conducted medical history inquiry, physical examination, diagnosis and treatment. All the surveys and tests were performed and assessed by the teachers who were blind to this study. After the students finished all curriculums, we distributed a questionnaire of specialty selection to all students again to assess whether teaching reform increases students’ interest in neurosurgery and medical student recruitment into neurosurgery.

### Statistical analysis

The differences between the two groups in gender, the student’s satisfaction with the course, and neurosurgery major selection in the pre- and post-curriculum were analyzed using Pearson’s chi-squared. The ages and scores of pre-class basic knowledge, self-perceived competence, satisfaction with the course, post-class test and clinical practice abilities were presented as the means ± standard deviations and analyzed by using Student’s t test. *P* < 0.05 was considered statistically significant. Statistical analyses were all performed using SPSS (version 22.0, IBM Corp.).

## Results

### Baseline characteristics of the students

A total of 120 fourth-year students in 4 classes from September 2020 to June 2021 were included in the study. There were 61 females (50.8%) and 59 males (49.2%), and the mean age of the students participating in the experiment was 24 years (range from 22 to 28 years). No student was lost to follow-up, and all the students completed the procedures of the study. There was no significant difference between the CBL-PBL group and the traditional model group in gender (*P* = 0.36), age (*P* = 0.11), or pre-curriculum basic knowledge scores (*P* = 0.71) (Table 1).

**Table 1:** Comparison of basic data between two groups

	CBL-PBL group (*N* = 60)	Traditional model group (*N* = 60)	T (χ^2^) value	*P* value
Sex				
Male (n, %)	28 (46.7%)	33 (55.0%)	0.83	0.36
Female (n, %)	32 (53.3%)	27 (45.0%)		
Age (years)	24.2 ± 1.4	23.8 ± 1.3	1.63	0.11
Pre-class basic knowledge score (point)	68.8 ± 7.9	69.3 ± 7.5	0.379	0.71

The total score for the pre-class exam is 100 points

### Comparison of self-perceived competence and satisfaction with the course between the two groups

After the course, we conducted a questionnaire on students’ self-evaluation and satisfaction with the course. As shown in Table 2, the item of learning motivation score in the CBL-PBL group was 4.2 ± 0.9, which was more than the 3.6 ± 1.0 score in the traditional model group (*P* = 0.004). The scores for the item of self-learning skills in the CBL-PBL group were 3.9 ± 1.0, higher than the 3.5 ± 1.1 score in the traditional model group (*P* = 0.019). The scores for the item of learning through a more targeted and more interesting approach in the CBL-PBL group were 4.2 ± 0.9, higher than 3.5 ± 1.0 in the traditional model group (*P* < 0.001). The scores on the item of basic knowledge mastery were 4.0 ± 1.0 in the CBL-PBL group, higher than the 3.6 ± 1.1 score in the traditional model group (*P* = 0.023). In the CBL-PBL group, the scores for the items of teamwork skills, clinical thinking ability, and clinician-patient communication skills were all higher than those in the traditional model group (*P* < 0.01, *P* < 0.01, and *P* = 0.009, respectively). In addition, the degree of satisfaction in the CBL-PBL group was also higher than that in the traditional model group (*P* = 0.007) (Table 3).

**Table 2:** Comparison of self-perceived competence between two groups

Item	CBL-PBL group (*N* = 60)	Traditional model group (*N* = 60)	T value	*P* value
Learning motivation (point)	4.2 ± 0.9	3.6 ± 1.0	2.92	0.004
Self-learning skills (point)	3.9 ± 1.0	3.5 ± 1.1	2.39	0.019
Learning more targetedlly and more interestingly (point)	4.2 ± 0.9	3.5 ± 1.0	3.62	< 0.01
Basic knowledge mastery (point)	4.0 ± 1.0	3.6 ± 1.1	2.31	0.023
Teamwork skills (point)	3.8 ± 1.1	3.1 ± 1.0	3.79	< 0.01
Clinical thinking ability (point)	4.1 ± 0.9	3.3 ± 0.9	4.60	< 0.01
Clinician-patient communication skills (point)	3.8 ± 0.9	3.4 ± 1.0	2.64	0.009

The maximum score for each item is 5 points

**Table 3:** Comparison of satisfaction with the course between two groups

Degree of satisfaction	CBL-PBL group (*N* = 60)	Traditional model group (*N* = 60)	χ2 value	*P* value
Very satisfied (n, %)	35 (58.3%)	18 (30.0%)	12.03	0.007
Satisfaction (n, %)	12 (20%)	13 (21.7%)		
General (n, %)	7 (16.7%)	19 (31.7%)		
Unsatisfactory (n, %)	6 (10%)	10 (16.6%)		

### Comparison of the post-class test scores between the two groups

In the CBL-PBL group, elementary knowledge scores were 79.2 ± 9.3, higher than 74.3 ± 10.0 in the traditional model group (*P* = 0.007), case analysis scores in the CBL-PBL group were 79.2 ± 6.9, more than 73.3 ± 7.8 in the traditional model group (*P* < 0.01), and the total scores of the CBL-PBL group were 79.2 ± 6.8, also significantly higher than the 73.8 ± 7.7 score in the traditional model group (*P* < *0.01*) (Table 4).

**Table 4:** Comparison of the post-class test scores between two groups

Item	CBL-PBL group (*N* = 60)	Traditional model group (*N* = 60)	T value	*P* value
Elementary knowledge (point)	79.2 ± 9.3	74.3 ± 10.0	2.76	0.007
Case analysis (point)	79.2 ± 6.9	73.3 ± 7.8	4.40	< 0.01
Total scores (point)	79.2 ± 6.8	73.8 ± 7.7	4.04	< 0.01

The maximum score for each item is 100 points

### Comparison of the clinical practice abilities between the two groups

After the course, we also conducted a clinical practice ability evaluation for the two groups of students. As shown in Table 5, in the CBL-PBL group, medical history collection scores, physical examination scores, diagnosis scores, and treatment scores were 19.3 ± 2.9, 16.5 ± 3.1, 19.0 ± 2.3, and 19.1 ± 2.4, respectively, more than 17.1 ± 3.3, 14.9 ± 3.0, 16.9 ± 2.5, and 17.3 ± 2.5 in the traditional model group (*P* < 0.01, *P* = 0.006, *P* < 0.01, and *P* < 0.01, respectively). In the CBL-PBL group, the total score was 73.9 ± 7.0, which was more than the 66.2 ± 6.5 in the traditional model group (*P* < 0.01).

**Table 5:** Comparison of clinical practice abilities between two groups

Clinical practice ability	CBL-PBL group (*N* = 60)	Traditional model group (*N* = 60)	T value	*P* value
Medical history collection (point)	19.3 ± 2.9	17.1 ± 3.3	3.99	< 0.01
Physical examination (point)	16.5 ± 3.1	14.9 ± 3.0	2.78	0.006
Diagnosis (point)	19.0 ± 2.3	16.9 ± 2.5	4.61	< 0.01
Treatment (point)	19.1 ± 2.4	17.3 ± 2.5	3.88	< 0.01
Total scores (point)	73.9 ± 7.0	66.2 ± 6.5	6.16	< 0.01

The maximum score for each item is 25 points, and for the total score is 100 points

### Neurosurgery specialty selection between the two groups of students pre- and post-curriculum

At pre- and post-curriculum, we surveyed the specialty selection of all the students. The results are presented in Table 6. In the CBL-PBL group, only 3.4% of the students chose a neurosurgery major at the beginning of the semester, while 13.3% of the students chose a neurosurgery major at the end of the semester; the difference was statistically significant (*P* = 0.048). In the traditional model group, 6.7% of students chose a neurosurgery major at the beginning of the semester, while 3.4% of students chose a neurosurgery major at the end of the semester; no significant difference was observed.

**Table 6:** Neurosurgery specialty selection between the two groups of students pre- and post-curriculum

	CBL-PBL group (*N* = 60)	Traditional model group (*N* = 60)	χ2 value	*P* value
At the beginning of the semester				
Neurosurgery (n, %)	2 (3.4%)	4 (6.7%)	NA	0.679
Other majors (n, %)	58 (96.7%)	56 (93.3%)		
At the end of the semester				
Neurosurgery (n, %)	8 (13.3%)	2 (3.4%)	3.93	0.048
Other majors (n, %)	52 (86.7%)	58 (96.7%)		

*NA* Not available

## Discussion

Although neurosurgery is popular among medical students in other countries, in China, it is still a challenge to recruit high-quality students into neurosurgery [20–23]. How to recruit high-quality students into neurosurgery in China is an important question that needs to be addressed. In this study, we use CBL and PBL integrated methods to implement teaching, which makes it easier for students to master neurosurgery knowledge and thus boosts their interest in neurosurgery compared to the traditional teaching method. Meanwhile, students’ self-confidence was significantly increased, and more students were inclined to choose neurosurgery as a career.

The traditional teaching method is the most efficient and economical way to deliver core knowledge and concepts [6, 19]. It is very suitable for teaching in large, basic medical classes, such as physiology, biochemistry, and tissue embryology, because these courses are focused on students’ mastery of knowledge points. However, in the teaching of clinical courses, such as internal medicine, surgery, gynecology, and pediatrics, more attention is given to the cultivation of students’ ability to integrate theory with practice and clinical thinking ability. The traditional teaching model is not suitable for the teaching of these courses. However, thus far, no standard teaching plan for clinical courses has been developed.

Most instructors are constantly exploring new teaching methods for clinical courses [15–19]. In this study, the results revealed that the scores of students’ self-evaluation, theoretical examination, and the students’ clinical application ability evaluation tests of those who received the CBL and PBL integrated methods were higher than those students who received the traditional teaching method, suggesting that the CBL and PBL integrated method is more suitable for neurosurgery teaching.

CBL and PBL integrated methods have been demonstrated to be better than the traditional teaching method in the teaching of other clinical courses. Liu et al. adopted CBL-PBL teaching for maxillary sinus floor augmentation, and better results were obtained in terms of academic knowledge acquisition, case analysis ability, and student satisfaction compared to the traditional teaching method [17]. Zhao et al. also demonstrated that in teaching about thyroid disease, CBL and PBL integrated methods improved residents’ and medical students’ performance and enhanced their clinical skills compared to the traditional teaching method [19]. However, some scholars’ research shows that student performance has not been improved with the new teaching method and that students prefer traditional lecture-style teaching [24, 25].

CBL is an active learning process. Students focus on the patient’s case, engage in scientific inquiry, self-guided learning, and collaboration with classmates, integrating theory into practice, developing clinical problems’ solving ability and critical thinking ability. PBL is an instructional approach that promotes students to integrate theory into practice and apply knowledge to develop viable solutions to some scheduled problems. It aims to help students develop their problem-solving abilities building upon their basic and clinical knowledge base [11–16].

**Fig. 3:**
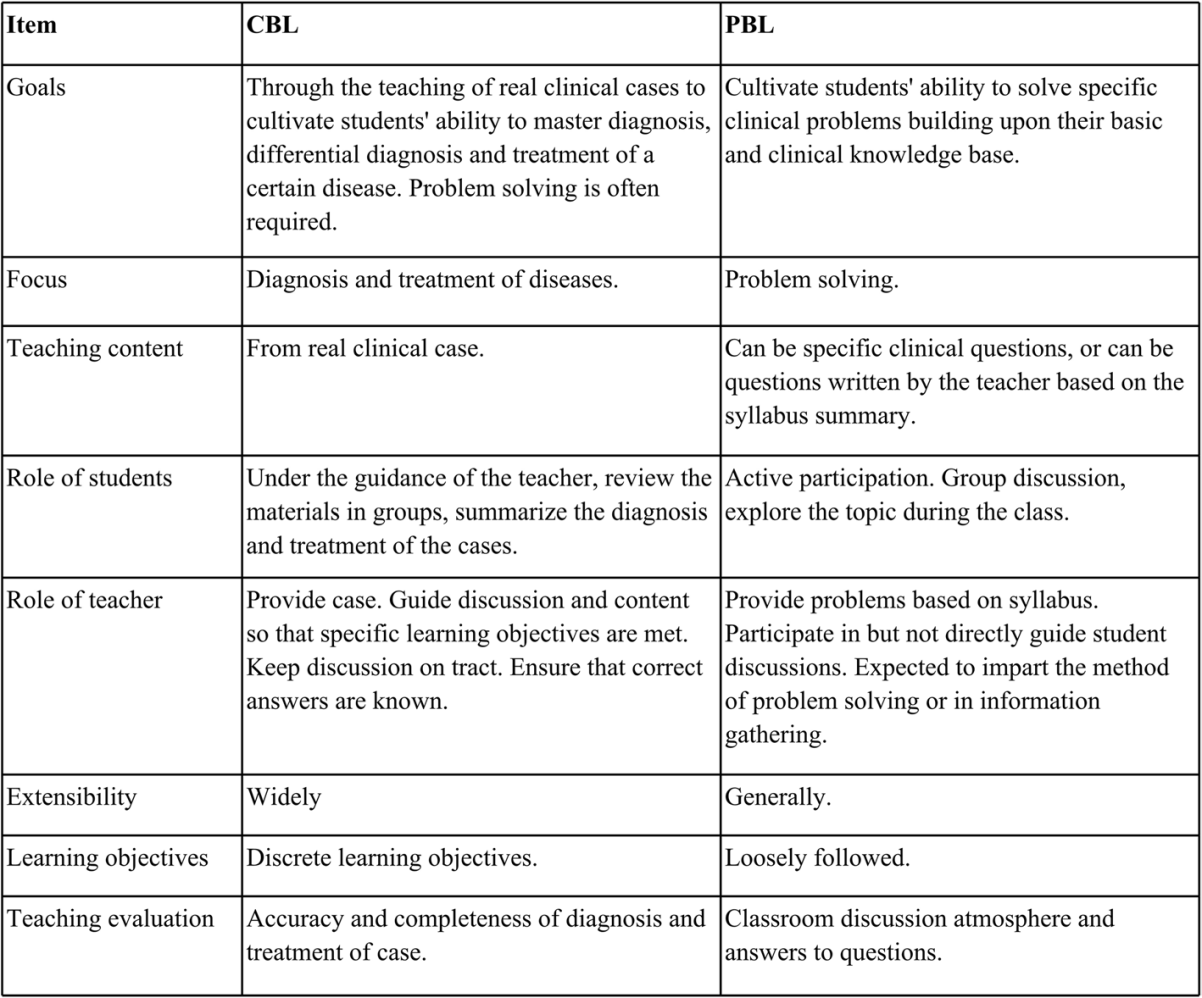
Differences between CBL and PBL in clinical teaching

Actually, CBL is considered a derivative of PBL, and the two are often confused (Fig. 3) [15, 16]. Srinivasan et al. pointed out that, unlike PBL, CBL often requires a certain basic theoretical knowledge of the subject [26]. Obviously, it is inadequate if CBL is used alone for the clinical teaching of undergraduates because they do not have theoretical knowledge of various fields. In addition, CBL pays more attention to the analysis of clinical cases, not just to the mastery of professional knowledge. However, PBL can make up for these shortcomings of CBL. As in this study, when assigning cases, we summarize the knowledge points that student need to master in problems and let students analyze cases based on the type of problem. The students’ clinical skills improved significantly, and it was easier for them to master theoretical knowledge.

Another advantage of CBL is that neurosurgery can be exposed to students early in the form of cases, and early exposure to neurosurgery contributes to medical student recruitment [27, 28]. In this study, students’ interests in neurosurgery increased through case teaching, and neurosurgery selection at the end of the semester was increased. However, the teaching methods of CBL and PBL integrated methods also have certain shortcomings. This teaching method is only suitable for small class teaching, which not only requires more neurosurgeons to participate in medical teaching but also requires neurosurgeons to have a great interest in teaching. Compared with the traditional teaching method, the PBL and CBL teaching methods require instructors to dedicate more time and energy.

There are several inevitable limitations in this study. First, this is a single-institution, small-sample study, and a multi-institution, large sample size study is needed. Second, this study cannot be completed in a double-blind manner because students may communicate privately. Third, although the teaching procedures of CBL and PBL integrated methods are uniform, the teaching style of each teacher in the traditional teaching model is different. Therefore, the results obtained may be biased. Last, teachers of other majors may also choose some new teaching methods, which may impact students’ major selection. This study did not capture these details.

## Conclusion

In conclusion, compared to the traditional teaching method, the CBL and PBL integrated method is more effective for improving medical students’ performance and enhancing their clinical capabilities when teaching neurosurgery. The students’ self-perceived competence and satisfaction with the course were obviously increased. Additionally, the CBL and PBL integrated methods effectively enhanced students’ interest in neurosurgery, potentially contributing to improving medical student recruitment into neurosurgery.

## Supplementary Information

**Additional file 1: Supplementary Table.** Original data for this study.

## Data Availability

All data generated or analyzed during this study are included in this published article [and its supplementary information files].

## Abbreviations

CBL: Case-based learning
PBL: Problem-based learning
